# Preferred Tempo and Low-Audio-Frequency Bias Emerge From Simulated Sub-cortical Processing of Sounds With a Musical Beat

**DOI:** 10.3389/fnins.2018.00349

**Published:** 2018-05-29

**Authors:** Nathaniel J. Zuk, Laurel H. Carney, Edmund C. Lalor

**Affiliations:** ^1^Department of Biomedical Engineering, University of Rochester, Rochester, NY, United States; ^2^Department of Neuroscience, University of Rochester Medical Center, Rochester, NY, United States; ^3^Del Monte Institute for Neuroscience, University of Rochester Medical Center, Rochester, NY, United States; ^4^Trinity Centre for Bioengineering, Trinity College Dublin, Dublin, Ireland

**Keywords:** auditory, rhythm, tempo induction, musical beat, biomimetic model

## Abstract

Prior research has shown that musical beats are salient at the level of the cortex in humans. Yet below the cortex there is considerable sub-cortical processing that could influence beat perception. Some biases, such as a tempo preference and an audio frequency bias for beat timing, could result from sub-cortical processing. Here, we used models of the auditory-nerve and midbrain-level amplitude modulation filtering to simulate sub-cortical neural activity to various beat-inducing stimuli, and we used the simulated activity to determine the tempo or beat frequency of the music. First, irrespective of the stimulus being presented, the preferred tempo was around 100 beats per minute, which is within the range of tempi where tempo discrimination and tapping accuracy are optimal. Second, sub-cortical processing predicted a stronger influence of lower audio frequencies on beat perception. However, the tempo identification algorithm that was optimized for simple stimuli often failed for recordings of music. For music, the most highly synchronized model activity occurred at a multiple of the beat frequency. Using bottom-up processes alone is insufficient to produce beat-locked activity. Instead, a learned and possibly top-down mechanism that scales the synchronization frequency to derive the beat frequency greatly improves the performance of tempo identification.

## Introduction

When we spontaneously tap our feet to music, we are “feeling the beat.” A musical beat is frequently defined by the effect it has on motor entrainment (Patel, 2010; London, 2012), and it is often identified as the fundamental level in the metrical hierarchy for keeping time (Lerdahl and Jackendoff, 1983). Many cultures have music with a beat, and the presence of beat-based music is highly related to communal dance (Savage et al., 2015). Clearly, perceiving the beat is key to the perception of music.

In many genres of music, musical beats often, but not always, occur at isochronous intervals (London, 2012). Previous models have simulated the perception of isochronous beats using an internal clock (Povel and Essens, 1985), pattern matching (Rosenthal, 1992; Parncutt, 1994), an internal resonator (van Noorden and Moelants, 1999), or a bank of neural oscillators (Large et al., 2015). These models often compute the beat frequency of discrete pulses, although a few have used annotated performances as input (ex. Rosenthal, 1992) or “onset signals” computed from cochlear-like filtering of audio signals (Scheirer, 1998; Large, 2000). Using electroencephalography and magnetoencephalography, it has been shown that cortical activity time-locks to the perceived beat for simplistic stimuli (Snyder and Large, 2005; Iversen et al., 2009; Nozaradan et al., 2011, 2012; Fujioka et al., 2012, 2015; Tierney and Kraus, 2015; Tal et al., 2017; but see Henry et al., 2017). Yet multiple stages of processing occur prior to cortical processing, each of which could affect the placement of musical beats.

Even for basic acoustic events, human subjects are biased to tapping to beats at inter-onset intervals of 500 to 700 ms (Parncutt, 1994), equivalent to a tempo range of 85 to 120 BPM. This range encompasses the “indifference interval” (Fraisse, 1963; London, 2012) for which subjects tap naturally at a regular rhythm (Semjen et al., 1998), discriminate tempi best (Drake and Botte, 1993), and can best replicate the duration of the interval (Stevens, 1886; Woodrow, 1934) (for review see Fraisse, 1963; Patel, 2010; London, 2012). This range also overlaps the range of tempi for a large proportion of dance music, which centers on 450 to 600 ms for intervals between beats, or equivalently 100 to 133 BPM (van Noorden and Moelants, 1999). However, an explanation for this optimal range of tempi is unclear. Motor entrainment plays a role in this bias since subjects tap naturally within this range, but it does not completely explain the optimization observed in studies that do not involve motor entrainment. Modulation tuning in the sub-cortical central nervous system would affect the synchronization strength of neural activity to isochronous acoustic events, which in turn could influence the preferred tempo.

Additionally, there is some evidence that our perception of musical beats is biased to certain ranges of audio frequencies. Listeners' ratings of “groove” in music, a subjective quality related to how much people want to move to the music, is correlated with the fluctuation in energy in low frequency (<200 Hz) and mid-frequency (400–1600 Hz) bands (Stupacher et al., 2016). Subjects also identify beats in piano ragtime music better when the left hand (lower frequency) is played alone than when the right hand (higher frequency) is played alone, although this could be due to the regularity of the left hand for this type of music (Snyder and Krumhansl, 2001). A low-frequency bias for beat timing could result from cochlear processing, where low frequencies cause a greater spread of excitation than higher frequencies (Hove et al., 2014), but these effects need to be disambiguated from cochlear delays that can produce similar biasing effects for simultaneous events (Wojtczak et al., 2017). For repeating “frozen” noise, where the noise signal was identical on each repetition, subjects focus on mid-frequency perturbations in the signal, between 300 and 2,000 Hz, when tapping along with the repetition (Kaernbach, 1993). Overall, while there does appear to be a frequency bias for time locking beats in music and repeating sounds, the exact frequency range of the bias, and the influence of subcortical processing on the bias, is still unclear.

Separately, several groups have developed “tempo-induction” algorithms that identify the tempo of musical recordings (for review see Gouyon et al., 2006; McKinney et al., 2007). These algorithms typically consist of three stages: identify onsets in the music, determine the pattern and speed of those onsets, and determine the tempo based on several representations of these factors (ex. Elowsson and Friberg, 2015). While some of these algorithms use processes that are similar to the auditory system (ex. Scheirer, 1998), none have been built on biomimetic models of auditory processing that simulate the neural activity produced by stages of auditory processing below the cortex. This processing is important because beat perception is based on the neural activity projected to the cortex. Both physiological modulation tuning and the inherent randomness of neural signals present in realistic auditory processing could affect beat perception in real music.

Here, we developed a model that determines the tempo of recordings of music based on the simulated neural activity of the auditory nerve and amplitude modulation tuning in the brainstem and midbrain. We hypothesized that physiologically plausible synaptic processing, which results in amplitude modulation tuning in the midbrain, can impose a preferred tempo near 100 BPM (London, 2012). We also hypothesized that innate processing in the auditory nerve can explain our low-audio-frequency bias for timing musical beats. Lastly, we quantify the strength of neural synchronization to musical beats in musical recordings and assess different ways in which the beat frequency may be inferred based on sub-cortical processing.

## Materials and methods

### Modeling

**Figure 1:**
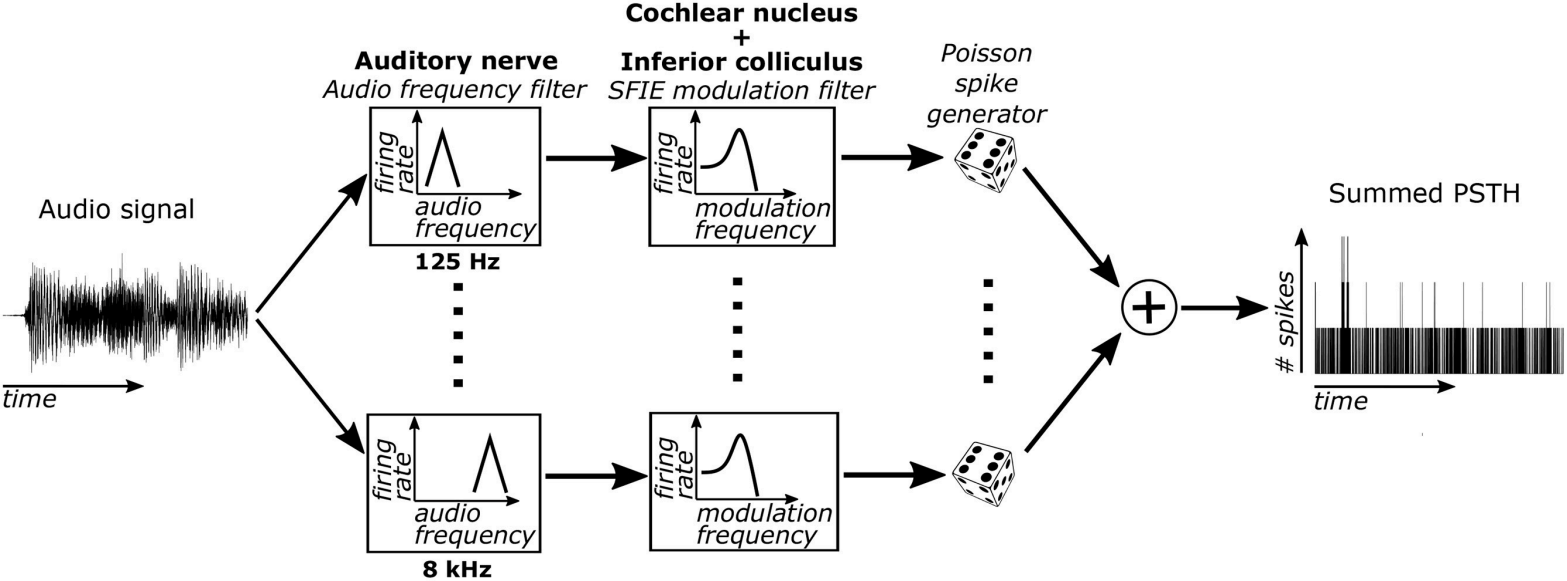
The model used to simulate sub-cortical neural activity consisted of three stages. First, the sound was filtered through 121 model AN fibers, each of which include bandpass filtering from the basilar membrane, compression due to the outer hair cells, and firing rate adaptation. Second, the output firing rates of these AN fibers were filtered using an SFIE model that simulated processing in the VCN and IC. Lastly, neural activity was simulated for each CF using the output time varying firing rate of the second stage. The simulated activity was then summed across CFs to get the summed PSTH.

Sub-cortical neural activity was simulated using a cascade of two biomimetic models for different stages of auditory processing. The sound input was converted to time-varying firing rates using a model of auditory-nerve (AN) fibers (Zilany et al., 2014) (Figure 1). Each AN fiber was tuned to a particular characteristic frequency (CF). The bandwidths of the model AN fibers matched human cochlear tuning (Shera et al., 2002). High-spontaneous-rate AN fibers were simulated with CFs from 125 to 8 kHz spaced every 0.05 octaves (121 fibers total). This model includes cochlear compression and firing rate adaptation (Zhang et al., 2001; Zilany et al., 2009). Our focus was on high-spontaneous-rate AN fibers because of their predominance in the auditory nerve (Liberman, 1978). Additionally, high-spontaneous-rate fibers alone can encode speech across a wide range of sound levels and in noisy environments (Carney et al., 2015), suggesting that they might also be especially important for encoding acoustic events relevant for musical beat perception.

**Figure 2:**
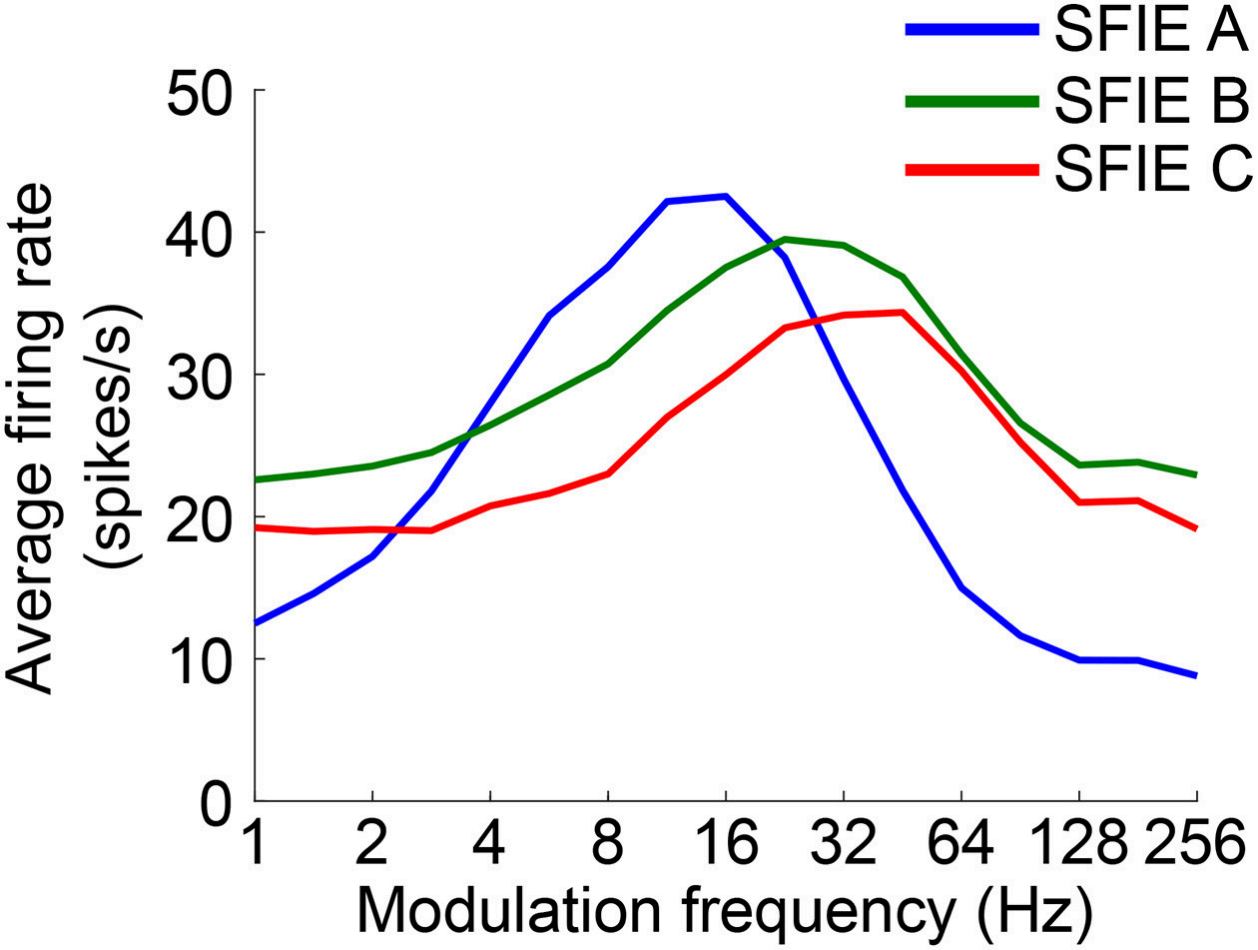
The rate modulation transfer functions for the three SFIE models we examined. The functions were computed by averaging the firing rate of the output of the SFIE model using a single input AN fiber (CF = 800 Hz) for 4 s of sinusoidally amplitude modulated broadband noise repeated 20 times. The parameters for each of the SFIE models can be found in Table 1.

The time-varying AN fiber firing rate was filtered using a model of synaptic processing in the ventral cochlear nucleus (VCN) and the inferior colliculus (IC) (Nelson and Carney, 2004; Carney et al., 2015). The model produces bandpass modulation sensitivity via two-stage same-frequency inhibition and excitation (SFIE), where the time constants, delays, and strengths of the inputs affect the neuron's best modulation frequency (Figure 2). The SFIE model also accentuates onset responses in the firing rate function that are akin to neural responses in the inferior colliculus or the medial geniculate body of the thalamus (Rouiller et al., 1981; Krishna and Semple, 2000; Bartlett and Wang, 2007; Nelson and Carney, 2007). We varied the SFIE model parameters (Table 1) to examine their effects on the strength of synchronization to a range of tempi.

**Table 1:** Parameters used for each two-stage SFIE model (see Nelson and Carney, 2004; Carney et al., 2015).

	**SFIE model parameters**				
	**τ_exc_ (ms)**	**τ_inh_ (ms)**	**S_inh_**	**d_inh_ (ms)**	**A**
VCN stage	0.5	2	0.6	1	1.5
IC A	5	10	1.1	2	6
IC B	2	6	1.1	2	2
IC C	1	3	1.5	2	2

*The parameters in the ventral cochlear nucleus (VCN) stage, the first stage of the model, were always used. The parameters for the second stage of the model, inferior colliculus (IC) model, was varied*.

For each of the 121 CFs, we randomly generated spike trains in response to each stimulus, assuming that the spike times obey an inhomogeneous Poisson process (Brown et al., 2002) with a time-varying rate parameter determined by the output of the SFIE stage. The spike trains across CF were then summed to form a post-stimulus time histogram (PSTH) for each response to a stimulus.

**Figure 3:**
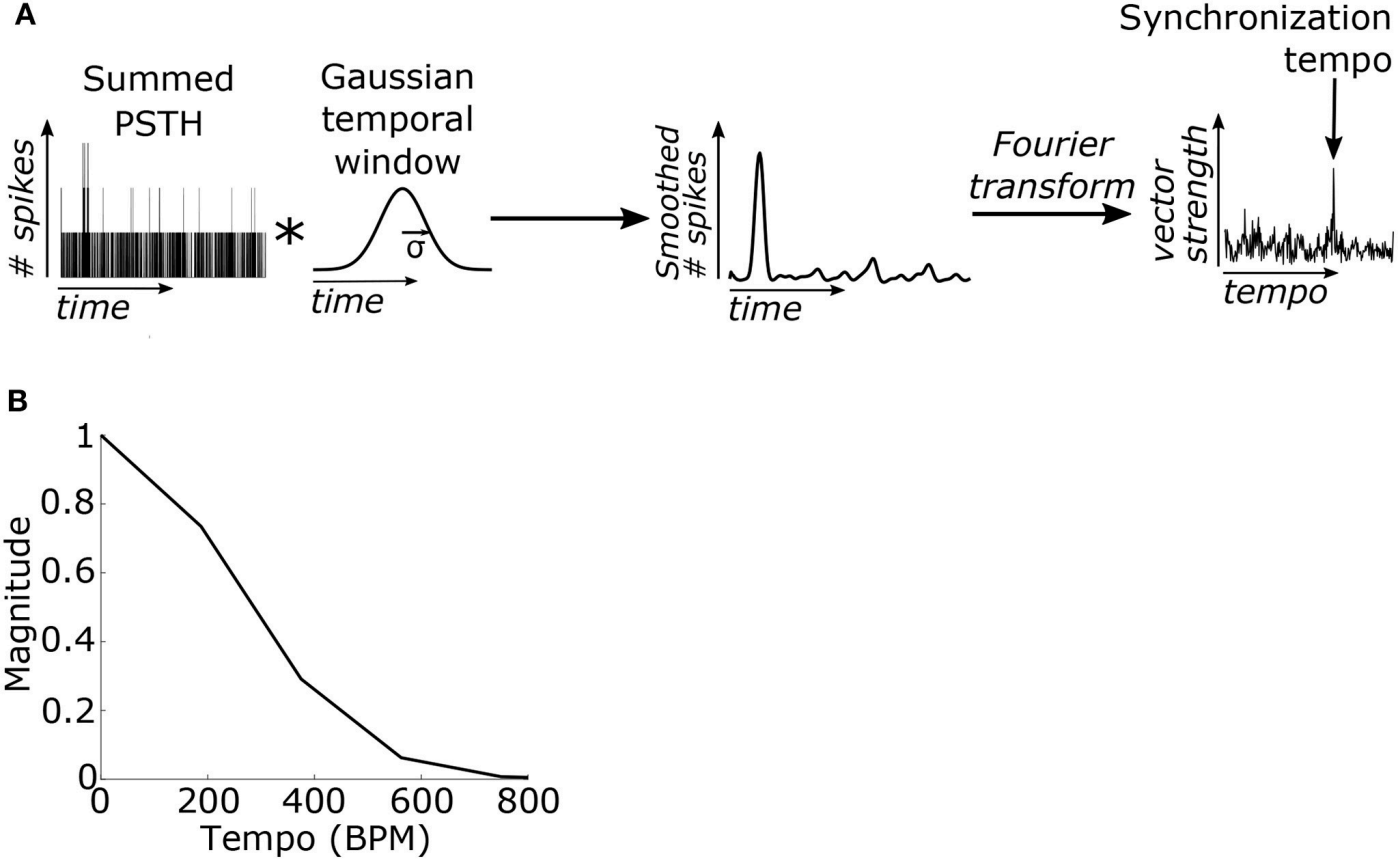
**(A)** The summed PSTH was convolved (represented by an asterisk) with a Gaussian-shaped temporal smoothing window with a standard deviation of 40 ms (see Materials and Methods). Then the Fourier transform of the smoothed PSTH was used to compute the vector strength of the neural activity, which quantifies the strength of synchronization, at each tempo. The “synchronization tempo” using this method was equal to the tempo with the peak vector strength between 30 and 600 BPM. **(B)** The Fourier transform of the temporal smoothing window. The temporal smoothing window smooths the PSTH and suppresses the vector strengths at high tempi.

We hypothesized that the beat frequency of the stimulus could be determined based on the phase-locking of the PSTH to the beat frequency. The PSTH was first filtered using a Gaussian-shaped temporal smoothing window. The shape of the window was based on prior results showing a Gaussian-like variation in performance for detecting events that deviate from isochronous intervals (Repp and Penel, 2002). Periodicities in the PSTH were then identified by taking the Fourier transform of the PSTH and normalizing by the average value of the PSTH (or the magnitude of the Fourier component at 0 Hz) (Figure 3A). This value is computationally identical to the “vector strength” which quantifies the synchronization strength of neural activity to a particular frequency (Goldberg and Brown, 1969). The model's “synchronization tempo” was the tempo where the vector strength was maximal.

In the Fourier domain the temporal smoothing window imposed a low-pass filter on the vector strength and thus suppressed the vector strength of fast tempi (Figure 3B). Several studies have demonstrated that the upper limit of the human perception of isochrony occurs at inter-onset intervals around 100 ms (for review see Repp, 2005; London, 2012). To enforce this upper limit, the standard deviation (σ) of the temporal window was empirically set to 40 ms because it was the minimum σ such that the vector strength for isochronous clicks at 600 BPM (inter-onset interval of 100 ms) was no larger than the vector strength for 100% jittered clicks at the same average rate (Supplementary Figure 1). The temporal window width of 40 ms was used for all SFIE models examined.

Throughout, all stimuli were set to 70 dB SPL and were up-sampled to a 100 kHz sampling rate, which was required for the AN fiber model. For stimuli that started or ended with a non-zero signal (for example, amplitude modulated noise), 15 ms raised-sine ramps were applied to the start and end of the stimulus.

### Stimuli and hypotheses for preferred tempo analyses

Stimuli were 10 s long and consisted of either 1 ms clicks (0.5 condensation followed by 0.5 ms rarefaction), sinusoidally amplitude modulated (SAM) broadband noise (0–50 kHz), square wave (SW) modulated broadband noise with a duty cycle of 50%, and raised-sine 100-ms-long tone pips with carrier frequencies of 250 Hz, 1 kHz, or 4 kHz. The tempo was varied from 30 BPM to 600 BPM in 30 BPM steps, and each stimulus was presented 10 times. The phase of the stimulus modulation was randomized for each presentation. The preferred tempo was determined for each type of stimulus using quadratic interpolation. To evaluate the effects of the SFIE model on this result, the analysis was repeated for each type of SFIE unit and also for the summed activity of the AN fibers alone.

Several studies have demonstrated that humans' ability to perceive and reproduce regular events is optimized for inter-onset intervals around 600 ms, corresponding to a tempo of 100 BPM (London, 2012). We hypothesized that the modulation filtering of the SFIE model and the temporal smoothing window could produce a vector strength maximum around 100 BPM. Additionally, Henry et al. (2017) showed that the strength of perceived musical beats is independent of the envelope of the stimulus. Based on this, we expected the tempo exhibiting the maximum vector strength (the “preferred tempo”) to remain the same irrespective of the stimulus being used.

### Assessing a frequency bias for tempo induction

To identify a frequency bias in tempo induction that could result from subcortical processing, we presented the model with stimuli consisting of two stimulus trains of 100 ms raised-sine tone pips presented at two different tempi (from the range 60 to 180 BPM) and two different frequencies (from the range 125 to 8,000 Hz) (an example stimulus can be found in Figure 6A). The tempi, frequencies, and phases of the two tones were randomly selected to generate 1000 different stimuli, and each stimulus was presented once. The frequencies of the two tone pips were spaced at least one octave apart to reduce AN adaptation effects (Zilany et al., 2009) that could produce cross-frequency forward masking. For each stimulus, we computed the normalized synchronization tempo (NST):

$$\begin{array}{l} NST=\;\frac{T_{sync}-T_{L}}{T_{H}-T_{L}} \end{array}$$

where *T*_*L*_ and *T*_*H*_ are the tempi for the low carrier frequency and high carrier frequency pulse trains, respectively, and *T*_*sync*_ is the synchronization tempo.

We expected the synchronization tempo to be close to the tempo of either the tone pips with the low-frequency carrier or the high-frequency carrier for most of the stimuli, resulting in an NST near either zero or one, respectively. Of those stimuli, we next examined how the other factors, the tempi of the two tone pips and their carrier frequencies, affected the NST. A logistic generalized linear model was fit to the NST values that were within ±0.08 of either zero or one (807/1000 trials) using fitglm in Matlab:

$$\begin{array}{l} P\left(NST=0|\mu \right)=\;\frac{e^{\mu }}{e^{\mu }+1} \end{array}$$

where:

$$\mu =\beta_{0}+\beta_{f_{L}}(\frac{f_{L}}{125})+\beta_{f_{H}}\log_{2}(\frac{f_{H}}{125})+\beta_{T_{L}}T_{L}+\beta_{T_{H}}T_{H}$$

where *f*_*L*_ and *f*_*H*_ are the carrier frequencies of the low and high frequency tone pips respectively, and the beta values quantify the linear dependence between each parameter and the probability that the NST equals one. If the NST was independent of the stimulus parameters, then the model should not do significantly better than a constant model (μ = β_0_). The significance of this difference was assessed using a likelihood ratio test. The significance of the individual coefficients in the model was also assessed using a likelihood ratio test comparing the full model to a reduced model with each component removed individually.

### Tempo induction of real music

Lastly, we examined how well this model could correctly identify tempi for two datasets of music: a “Ballroom” dataset of 685 clips of ballroom dance music (after removing exact and recording replicates, see Sturm, 2014), and a “Songs” dataset of 465 clips of music from a wide variety of genres and cultures, including some dance music (Gouyon et al., 2006). These datasets are standards for assessing the performance of tempo-induction and beat-detection algorithms (Gouyon et al., 2006; McKinney et al., 2007). We determined the synchronization tempo based on the tempo between 30 and 600 BPM with the maximum vector strength. Throughout, the synchronization tempo was identified as correct if it fell within ±8% of the ground truth tempo (standard for the MIREX tempo induction competition, see McKinney et al., 2007).

### Computing the tempo using a classifier

Often, the peak vector strength occurred at a multiple of the ground truth tempo rather than at the actual ground truth tempo. One possibility is that we “feel the beat” for every 2–4 events depending upon the speed of the music (Parncutt, 1994; London, 2012). Additionally, we may be using the pattern of events in the music, or the “rhythm”, to determine the beat frequency, since beat perception is affected by rhythm (Povel and Essens, 1985; Parncutt, 1994). To understand the importance of speed and rhythm on tempo induction, we used regularized multi-class linear discriminant analysis (mcLDA) (fitcdiscr.m in Matlab, other classification algorithms did not perform as well) to develop two different classifiers that identify the “scaling factor” equal to the ratio of the synchronization tempo to the ground truth tempo, either 1, 2, 3, or 4. The first classifier used the synchronization tempo alone to classify the scaling factor; faster synchronization tempi were more likely to have higher scaling factors. For the second classifier, we reasoned that, if the model neurons were synchronizing to events in the music, then the rhythm of the music could be quantified by the number of times certain intervals appear between simulated spikes. The within- and across-CF interspike interval (ISI) histogram for the summed neural activity was computed using the autocorrelation of the summed PSTH, and the “ISI ratio” for a particular interval was computed by summing the ISIs within a 20 ms rectangular window surrounding the interval and dividing by the total number of ISIs between 0.1 and 30 s. The ISI ratio was computed for ISIs at the following multiples of the event period: 1/16, 1/12, 1/9, 1/6, 1/4, 1/3, 1/2, 2/3, 3/4, and 1. All stimuli from both datasets were included in this analysis, and the ratios were rounded to closest integer between 1 and 4. This classification procedure was repeated for 1000 random re-samplings of the stimuli, selecting 75% of the stimuli for training and 25% for testing. We determined whether the second classifier performed significantly better than the first by testing the null hypothesis that the distribution of differences in performance between the two classifiers for the 1000 re-samplings was no greater than 0.

## Results

### Dependence of model vector strength on stimulus tempo

Firstly, we examined if the vector strength of the model PSTH was maximal over a specific range of tempi. We hypothesized that sub-cortical processing could contribute to this biasing, which has been observed around 100 BPM. The vector strength as a function of tempo was computed using three different midbrain models (Table 1) that were tuned to different best modulation frequencies (Figure 2). For comparison, the vector strength was also computed based on the unfiltered summed AN fiber output.

**Figure 4:**
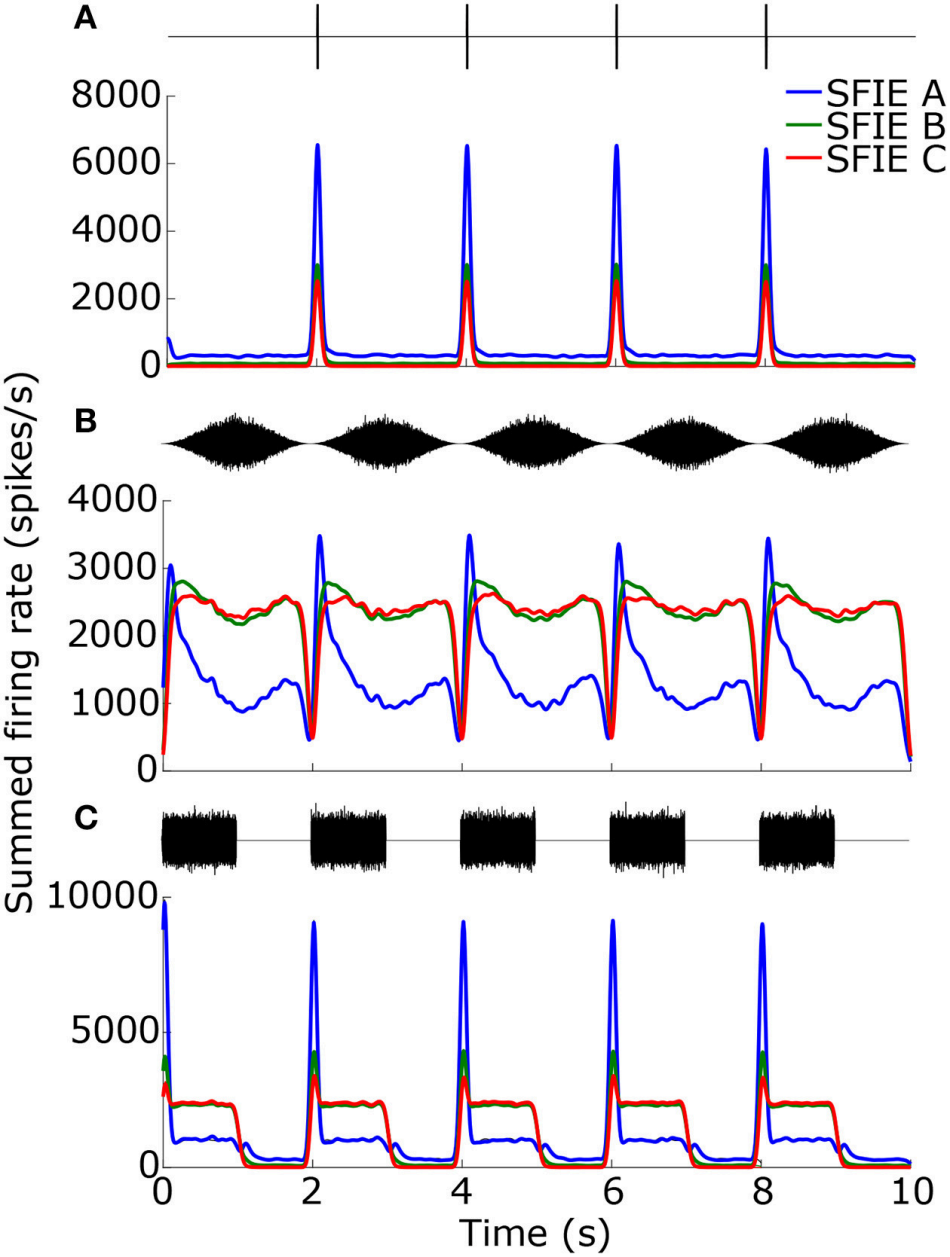
Firing rates for the different SFIE models in response to 1 ms clicks **(A)**, SAM broadband noise **(B)**, and SW noise **(C)** at 30 BPM (SFIE A: blue, SFIE B: green, SFIE C: red). The corresponding stimulus is shown above each plot of the firing rate. All stimuli were presented at 70 dB SPL. The firing rates were summed across CF and averaged across 10 repetitions of each stimulus with different noise tokens. Spontaneous firing during silences **(A,C)** and saturating firing rates during continuous noises **(A,B)** contributed to a falloff in vector strength at lower tempi (see Figure 5).

While the temporal smoothing window suppressed vector strengths at high tempi (Figure 3B), there was also a reduction in vector strengths at low tempi due to an intrinsic property of the auditory nerve model. Figure 4 shows examples of the summed firing rate across CF for different SFIE models, which was the input to the Poisson spike generator (Figure 1). For click trains at 30 BPM (Figure 4A), SFIE model A generated the largest firing rates in response to a click, but it also produced the highest spontaneous rate, resulting in the lowest vector strength of the three midbrain models. For SAM noise (Figure 4B), the firing rates of high-spontaneous rate AN fibers saturated at moderate sound levels, resulting in saturating SAM responses for moderate to high SPLs which reduced their synchronization strength (see also Joris et al., 2004). The saturating responses were maintained for the models with high peak modulation frequencies, SFIE B and C. In contrast, SFIE A showed a stronger onset response during the rising phase of the stimulus modulation followed by a reduction in firing during the rest of the cycle of the modulation. As a result, SFIE A had a larger vector strength than the other two models. For SW noise (Figure 4C), the response for model SFIE A showed both a suppression of sustained firing as well as high spontaneous firing.

**Figure 5:**
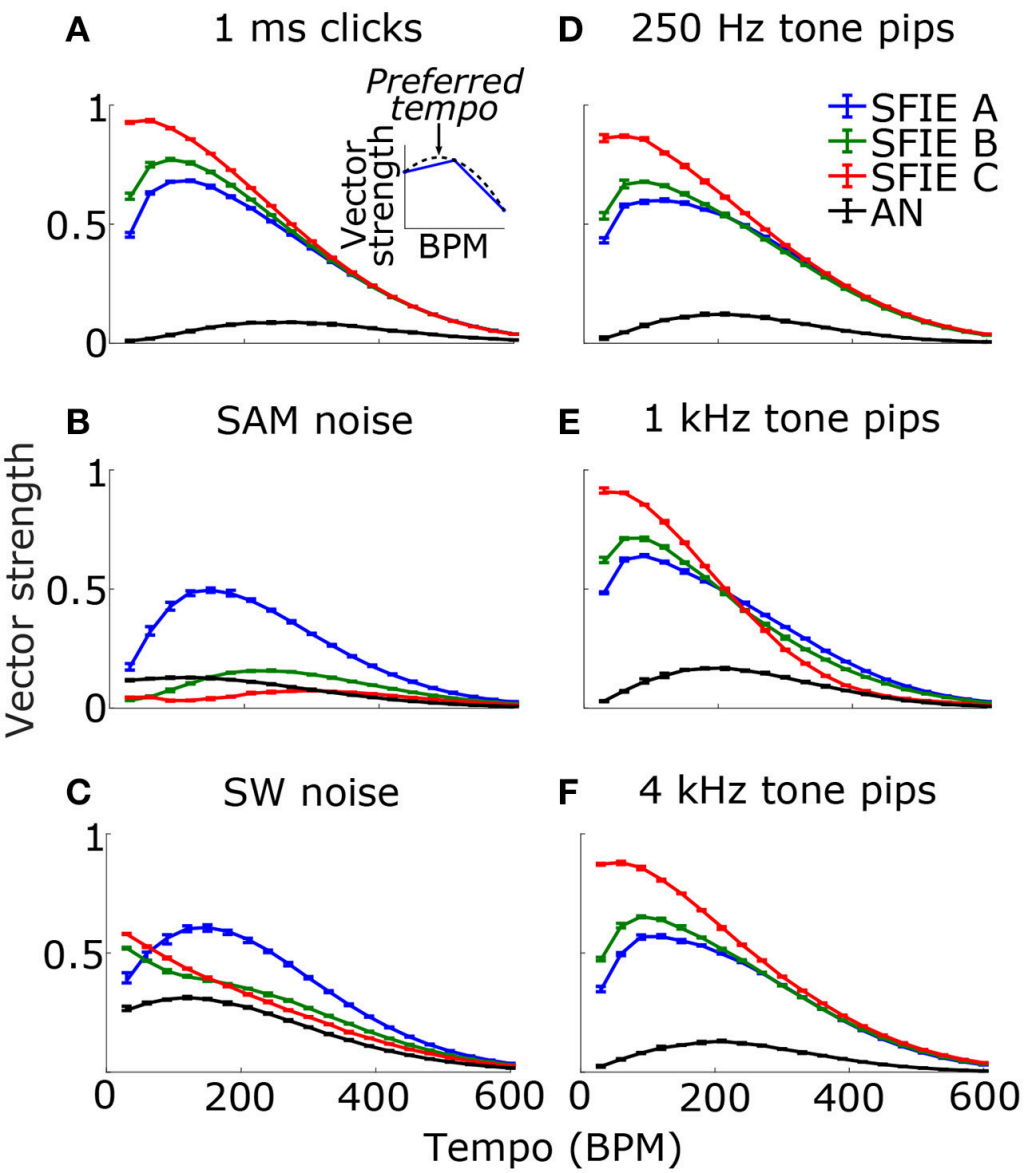
Vector strength as a function of tempo in response to 1 ms clicks **(A)**, SAM broadband noise **(B)**, SW broadband noise **(C)**, and tone pips with carrier frequencies of 250 Hz **(D)**, 1 kHz **(E)**, and 4 kHz **(F)**. The vector strengths for the different SFIE models are color coded identically to Figure 4. Error bars designate interquartile ranges for 10 repetitions of each stimulus. The vector strength using the AN fiber activity alone, without an SFIE stage, is also shown in black. SFIE model A consistently produced peak vector strengths within the range of tempi typically associated with the “indifference interval” (around 100 BPM) and overlapping the range of tempi for dance music (van Noorden and Moelants, 1999). The preferred tempos were determined by quadratic interpolation. The black dashed line in the inset in **(A)** shows the quadratic fit to the points surrounding the maximum vector strength for SFIE A. The preferred tempo is equal to the peak of the quadratic fit. Preferred tempos and peak vector strengths are quantified in Table 2.

Across a wide variety of stimuli (clicks, SAM noise, SW noise, tone pips), SFIE A consistently produced preferred tempi between 86 and 150 BPM (Figure 5, peak values summarized in Table 2). In contrast, peak vector strengths occurred at a much wider range of tempi for the other two SFIE models and for the AN fiber activity. Since human perception of musical beats is invariant to the envelope of the stimulus (Henry et al., 2017), these results strongly suggest that neurons with long excitatory and inhibitory synaptic time constants are important for musical beat perception and responsible for biasing the preferred tempo around 100 BPM. Such neurons would produce strong onset firing and reduced sustained firing necessary for creating salient beats. We also found empirically that vector strengths were larger for musical recordings using SFIE A than the other two models (Supplementary Figure 2). For these reasons, SFIE A was used when simulating sub-cortical neural activity in the following experiments.

**Table 2:** Preferred tempi (upper) and peak vector strengths (lower) are shown for each stimulus and SFIE model, including the summed AN fiber output without the SFIE model applied (see Figure 5).

	**Preferred tempo (BPM)**						
	**Peak vector strength**						
	**Clicks**	**SAM** **noise**	**SW** **noise**	**Tone pips**			
				**250 Hz**	**1 kHz**	**4 kHz**	**Average ± st dev**
SFIE A	111	146	142	112	86	106	117 ± 23
	0.68	0.50	0.61	0.60	0.64	0.57	0.60 ± 0.06
SFIE B	94	223	30	87	76	98	101 ± 64
	0.77	0.16	0.52	0.68	0.72	0.65	0.58 ± 0.23
SFIE C	51	299	30	56	30	52	86 ± 105
	0.94	0.07	0.58	0.87	0.91	0.88	0.71 ± 0.34
AN fibers	267	117	120	204	201	206	186 ± 58
	0.09	0.13	0.31	0.12	0.17	0.13	0.16 ± 0.08

*Maxima were computed using quadratic interpolation*.

### Dependence of the synchronization tempo on stimulus audio frequency

There is some evidence that human perception of musical beats may be biased to particular frequency ranges, but the strength of this effect and the underlying mechanism are unclear. We hypothesized that subcortical processing may produce a frequency bias for tempo induction. Specifically, when multiple carrier frequencies are present with temporal modulations at distinct tempi, we expected the synchronization tempo to equal the tempo of the lowest carrier frequency.

**Figure 6:**
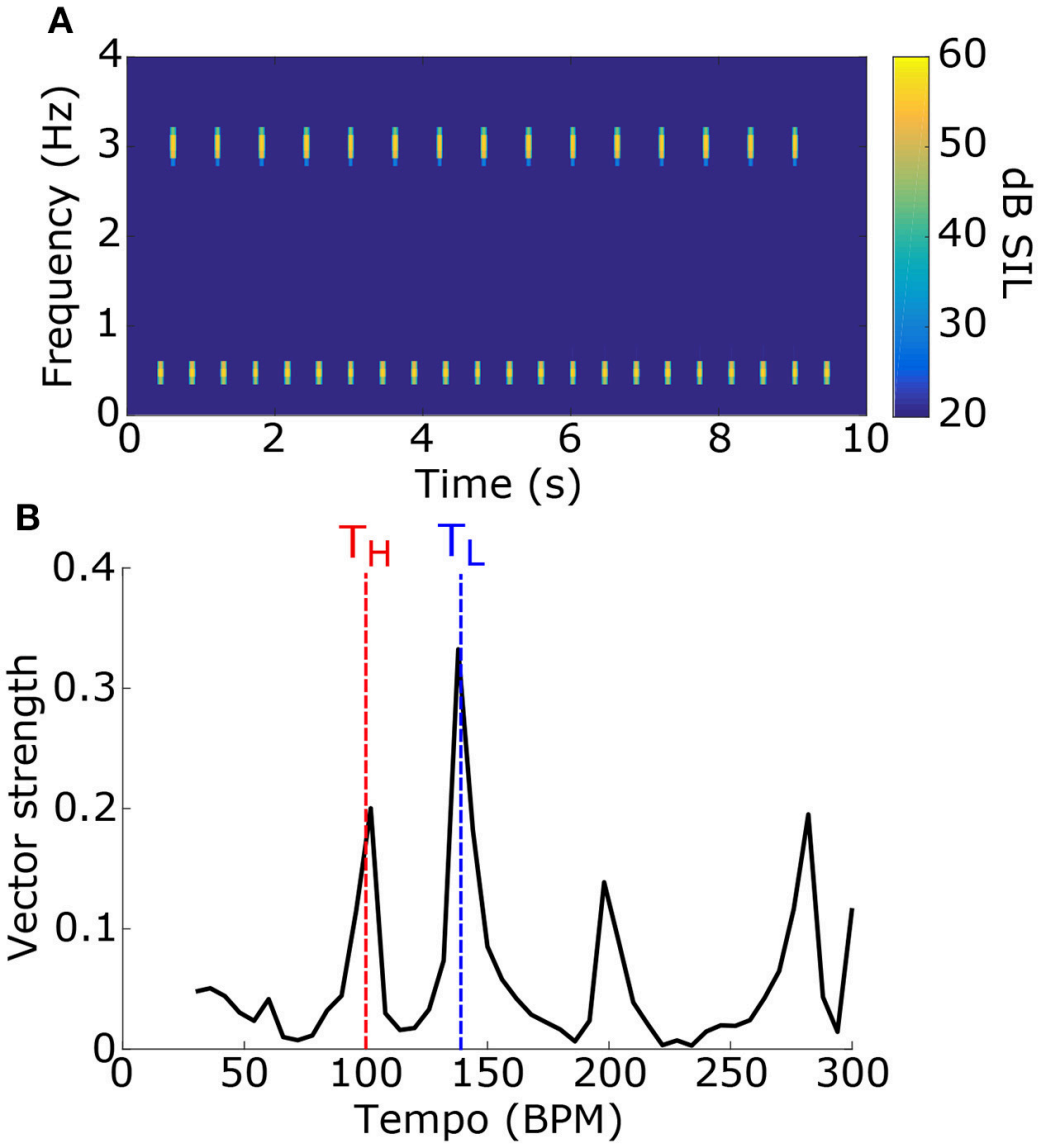
**(A)** To test for a frequency bias in tempo induction, stimuli consisted of two sets of tone pips at two different carrier frequencies and different tempi. An example stimulus power spectrogram is shown (tone 1: f_L_ = 500 Hz, T_L_ = 140 BPM; tone 2: f_H_ = 3 kHz, T_H_ = 100 BPM; phase = 0 for both). **(B)** The vector strength as a function of tempo for the stimulus in **(A)** is shown. Dashed lines mark the tempi for the tone pips with the low-frequency carrier (blue) and the high-frequency carrier (red). The synchronization tempo was 138 BPM and the NST was 0.05, indicating that it is close to T_L_.

**Figure 7:**
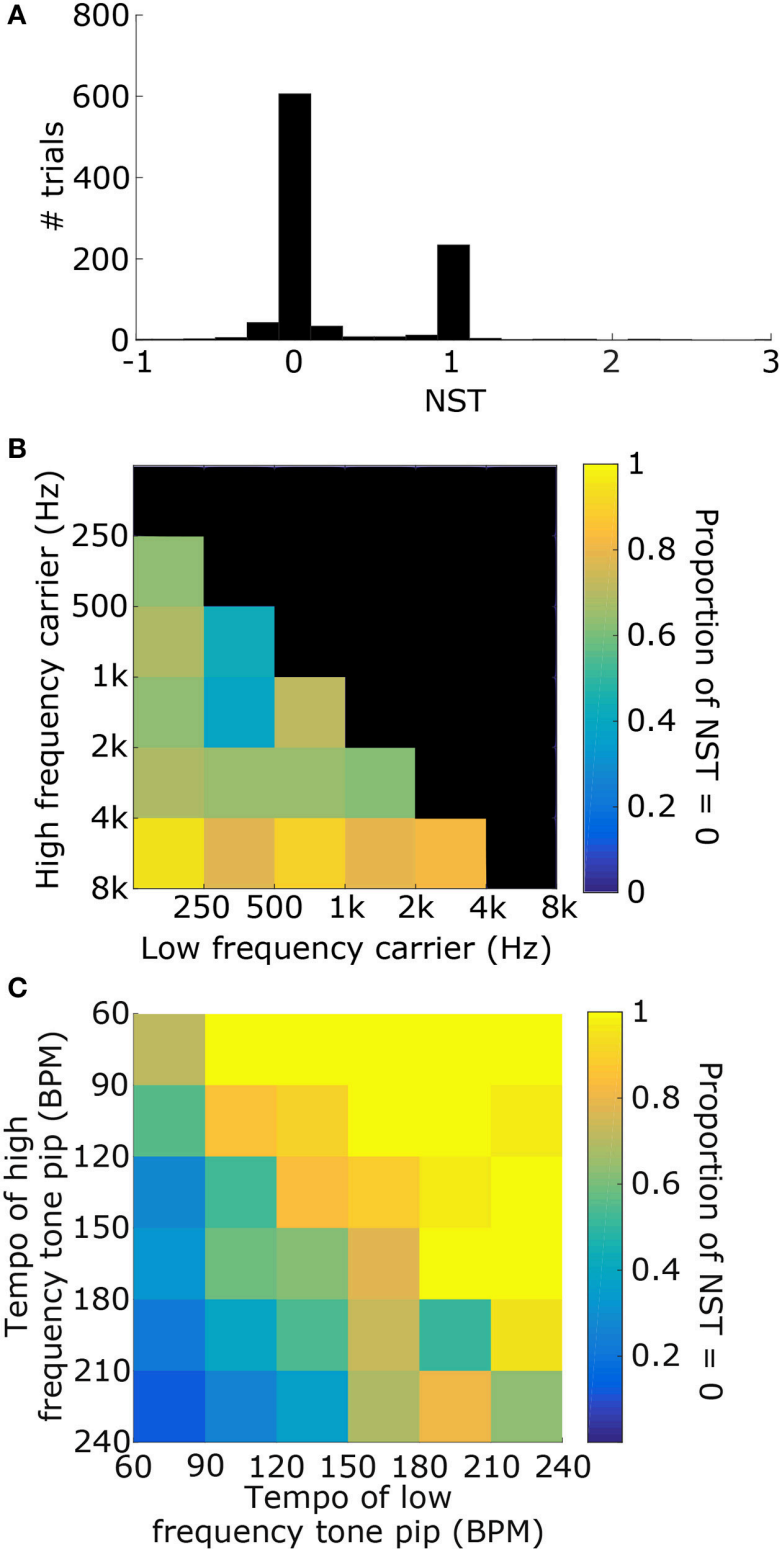
**(A)** Distribution of the NSTs for all 1000 randomly generated stimuli consisting of two tone pips. An NST of 0 means that the synchronization tempo is equal to T_L_. An NST of 1 means that the synchronization tempo is equal to T_H_. On average, the synchronization tempi were closer to T_L_. **(B)** Proportion of trials with NST = 0 with respect to the carrier frequencies of the stimulus. Each bin shows the marginal probability given f_L_ and f_H_. **(C)** Proportion of trials with NST = 0 with respect to the tempi T_L_ and T_H_, plotted similarly to **(B)**.

1000 stimuli were generated, consisting of two tone pips with carrier frequencies, tempi, and phases that were selected randomly (see Figure 6A for example). For each stimulus, the synchronization tempo was normalized relative to the tempos of the two tone pips to get the NST (Figure 6B). An NST of zero means that the synchronization tempo was closer to the tempo of the tone pip with the low-frequency carrier, and an NST of one means that it was closer to the tempo for the high-frequency carrier. 80.7% of the stimuli produced NSTs that were within ±0.08 of zero or one (Figure 7A). There were significantly more stimuli that produced NSTs near zero than near one (Chi-squared test: χ^2^ = 149, *p* < 0.001). On average, synchronization tempi were biased to lower audio frequencies.

The distribution of NSTs, however, also varied with the carrier frequencies (Figure 7B) as well as the tempi of the tone pips (Figure 7C). Each showed a monotonic relationship with the proportion of NSTs equal to zero. To quantify these dependences and assess their significance, we fit a logistic generalized linear model to the individual NSTs with the low-frequency carrier (f_L_), high-frequency carrier (f_H_), and the tempi of those tone pips (T_L_ and T_H_ respectively) as dependent variables (see Materials and Methods). We found that the generalized linear model fit significantly better than a constant model (Likelihood ratio test: χ^2^ = 530, *p* < 0.001), meaning that the carrier frequencies and tempi had a significant effect on the NST relative to the average bias observed initially (Figure 7A). Specifically, the NST was significantly dependent on f_H_ (β_fH_ = 1.39, χ^2^ = 78, *p* < 0.001) and both tempi (T_L_: β_TL_ = 0.043, χ^2^ = 253, *p* < 0.001; T_H_: β_TH_ = −0.034, χ^2^ = 193, *p* < 0.001). The effect of f_L_ was not significant (β_fL_ = −0.033, χ^2^ = 0.08, *p* = 0.77).

Overall, synchronization tempi were biased to the tempo for the tone pips with the lower carrier frequency, but the biasing was weakest when the interfering modulations from the higher carrier frequency was close to the lower carrier frequency. Both low-CF and high-CF responses resulted in similar vector strengths for broadband stimuli with tone-pip-like modulations, suggesting that the biasing observed here was due to the spread of excitation in the basilar membrane and not due to differences in the response properties of different CFs (Supplementary Figure 3). However, the tempi of the tone pips had a stronger influence on the synchronization tempo than the carrier frequency, and the synchronization tempo was more likely to equal the fastest tempo. This was contrary to our earlier finding that the vector strength was maximized around 100 BPM for salient, isochronous stimuli. When multiple competing modulations are present in complex stimuli, the faster modulations dominate in the summed synchronized activity, primarily because faster modulations produce more events and are more likely to mask slower modulations (Supplementary Figure 4).

### Tempo induction of real music

**Figure 8:**
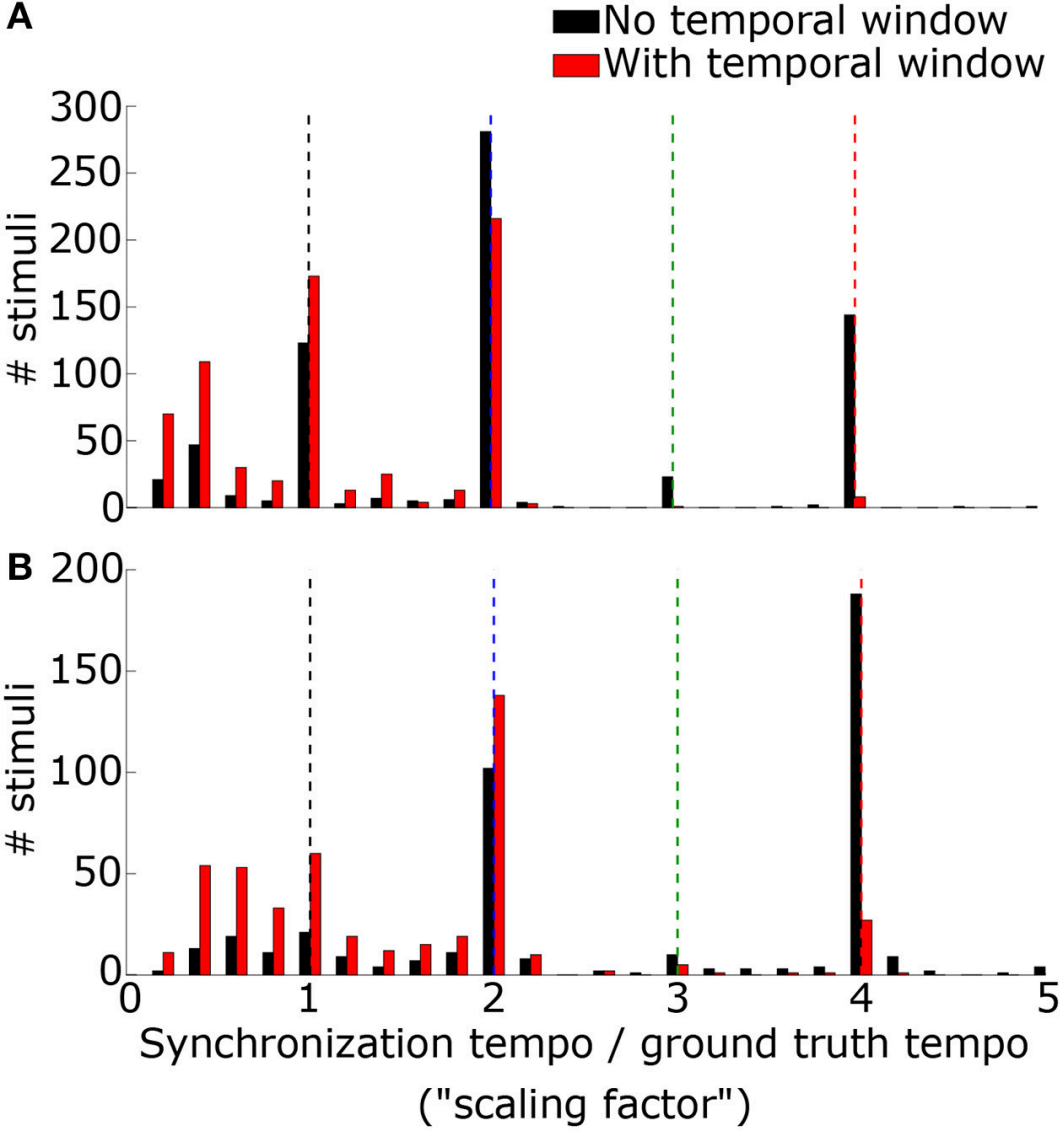
The histogram of the ratio between the synchronization tempo and the ground truth tempo is plotted for the Ballroom dataset **(A)** and the Songs dataset **(B)** without the temporal Gaussian window applied (black) and with the temporal Gaussian window (red). Colored dashed lines mark the scaling factors of 1x (black), 2x (blue), 3x (green), and 4x (red).

We lastly evaluated tempo-induction performance using two datasets widely used for testing tempo-induction algorithms (Gouyon et al., 2006): a “Ballroom” dataset of 685 ballroom dance music clips, and a “Songs” dataset of 465 songs from a wide variety of genres. For each stimulus the synchronization tempo was computed and compared to the ground-truth tempo for the recording. The synchronization tempo was equal to the ground-truth tempo for only 19.9% of the stimuli (25.0% for ballroom, 12.4% for songs) (Figure 8). More often, the synchronization tempo was twice the ground-truth tempo (31.7% for ballroom, 28.8% for songs, 30.5% overall).

**Figure 9:**
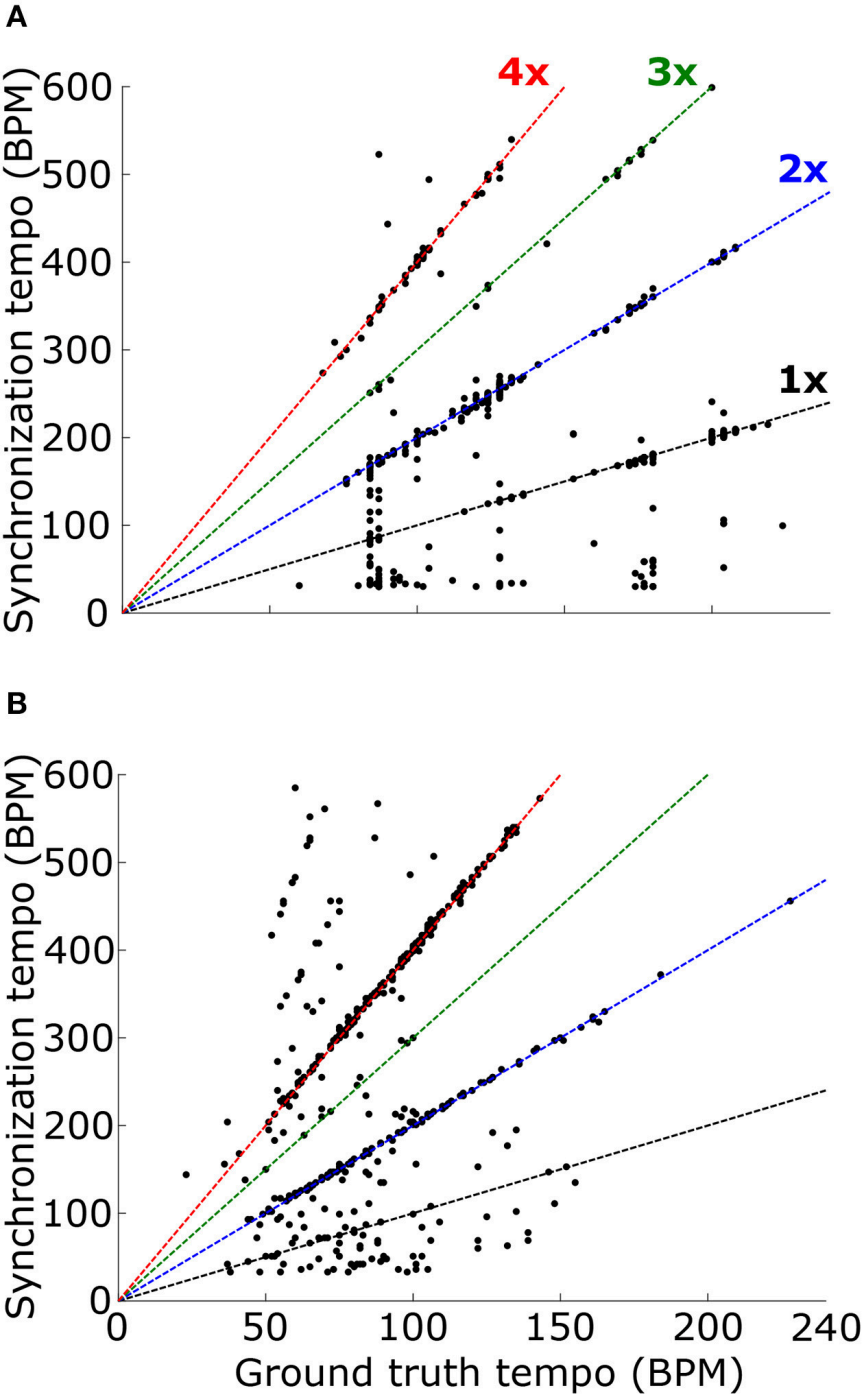
Synchronization tempo using the vector strength of the summed PSTH without the temporal smoothing window is plotted as a function of the ground truth tempo for the Ballroom dataset **(A)** and the Songs dataset **(B)**. Dotted lines mark the slopes corresponding to scaling factors 1–4, as in Figure 8. For the combined datasets (1163 stimuli total), 75.5% of the synchronization tempi fell within ±8% of these four slopes.

When the PSTH was not smoothed with the temporal smoothing window, fewer synchronization tempi were equal to the ground-truth tempo (18.0% for ballroom, 3.9% for songs, 12.2% overall) (Figure 8). However, most of the synchronization tempi occurred at a multiple of the ground-truth tempo: 75.5% of the stimuli produced synchronization tempi at 1-4x the ground truth (81.8% for ballroom, 66.2% for songs) (Figure 9). This accounted for 25.1% more of the stimuli than the number that had synchronization tempi at 1-2x the ground truth after smoothing the PSTH.

Thus, while the temporal smoothing window suppresses faster synchronous activity by low-pass filtering the PSTH, it does not unearth a subharmonic peak in vector strength at the true beat frequency of the music. Instead, the model's synchronized activity at a multiple of the ground truth tempo may serve as a reference for determining the actual tempo of the music.

### Scaling the synchronization tempo

**Figure 10:**
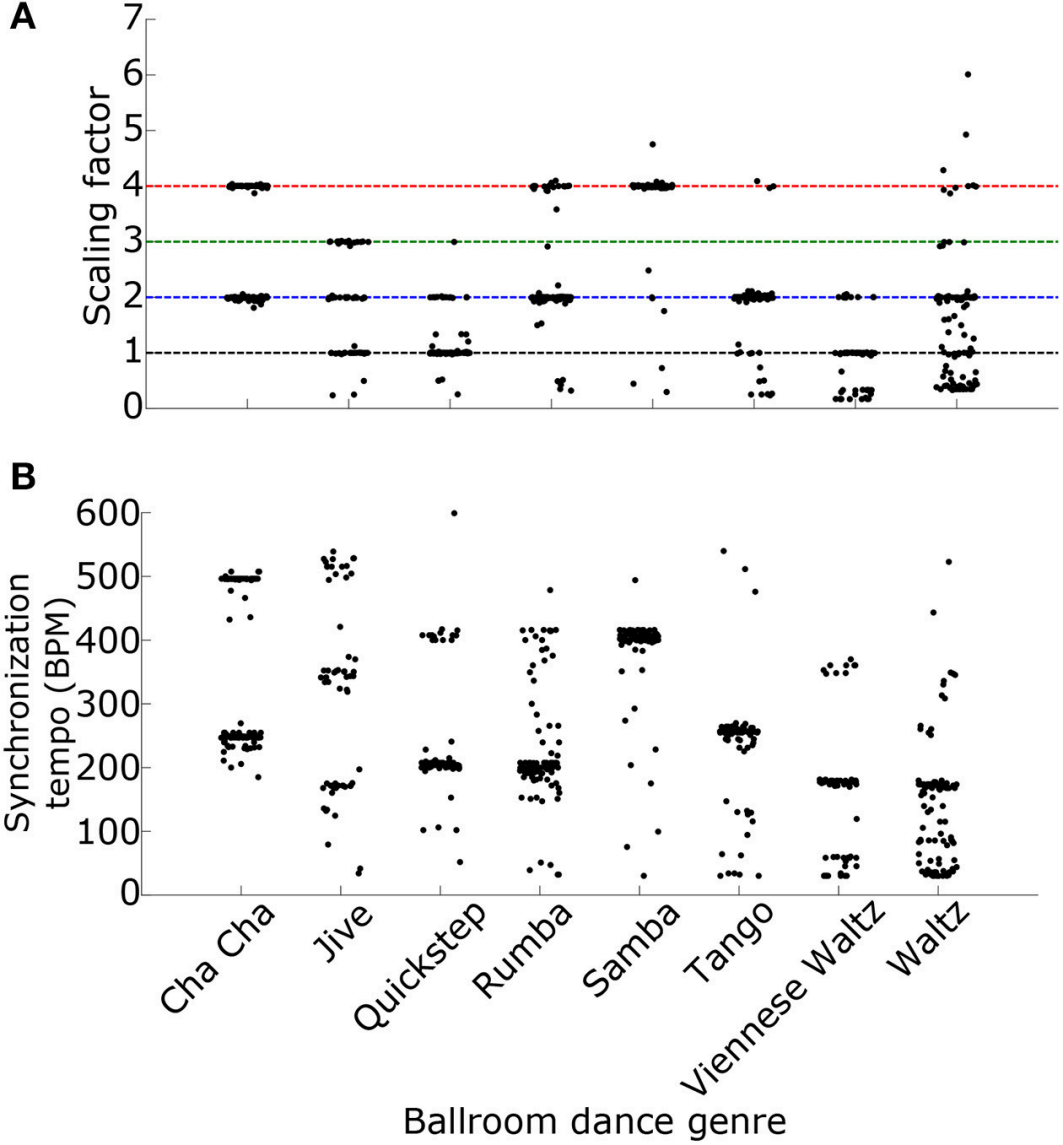
**(A)** The ratios of the synchronization tempo to the ground truth tempo (the “scaling factors”) and **(B)** the synchronization tempi for the 685 Ballroom stimuli are plotted as a function of the ballroom dance genre. Colored dashed lines mark the ratios 1–4, as in Figures 8, 9. Synchronization tempo and the scaling factor both depend upon the genre of the ballroom dance music.

Why is the most synchronous activity at a multiple of the ground truth tempo? One possibility is that the synchronous activity occurs at the “event frequency” of the music, a higher tempo than the beat frequency, such as the frequency of notes played by an instrument or the frequency of drum hits (London, 2012, see also Ding et al., 2017 for a similar result using the modulation spectrum). Indeed, we found that the ratio of the synchronization tempo to the actual tempo, the “scaling factor,” depended upon the genre of the ballroom dance music, suggesting that the relationship between the synchronization tempo and the actual tempo may depend upon the rhythm of the music (Figure 10A). Alternatively, the relationship between the event frequency and the tempo could depend upon the speed of events. As the speed of the events increases, the event frequency would need to be divided by a larger scaling factor in order to get the correct tempo. Because different ballroom dance genres can be qualitatively characterized by different speeds (for example: tango is slower than samba), the event frequencies may also be dependent upon genre. Indeed, we found that the synchronization tempo was dependent upon the genre of the music (Figure 10B). Whether the scaling factor is dependent upon the speed or the rhythm of the events, it should be possible to simply divide the synchronization tempo by a scaling factor in order to get the actual beat frequency of the music.

**Figure 11:**
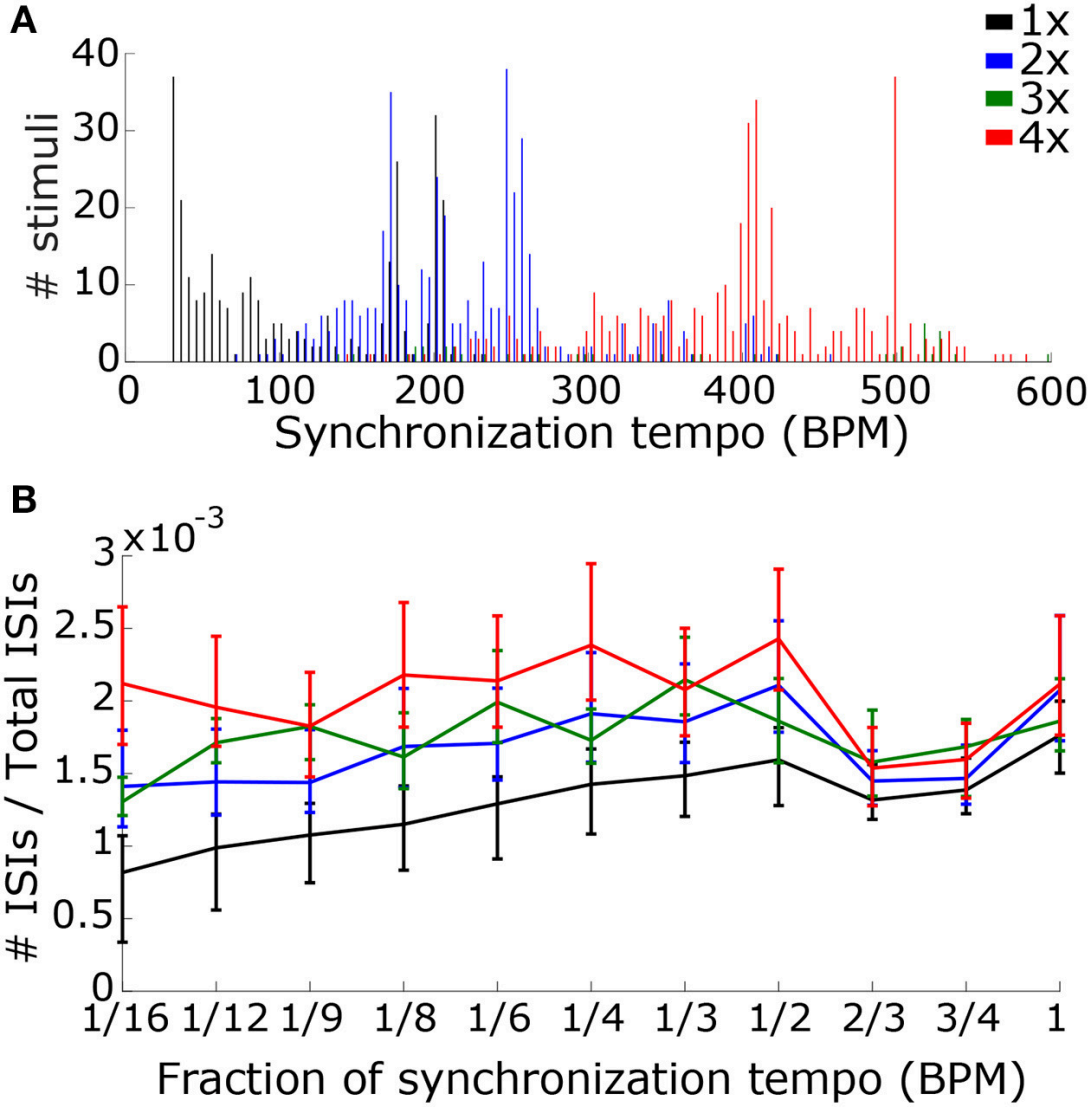
In order to determine the scaling factor, we created two classifiers that used speed and rhythm information in the summed PSTH. **(A)** The first classifier used the speed alone, quantified by the synchronization tempo. A distribution of synchronization tempi for each scaling factor is shown. **(B)** The second classifier used both speed and rhythm. Rhythm was quantified by the ISI ratios (the number of ISIs at a particular interval divided by the total number of ISIs) at intervals corresponding to fractions of the synchronization tempo. The median and interquartile range of the ISI ratios for each fraction is shown for each scaling factor.

To determine the scaling factor for each stimulus we used mcLDA to design two classifiers (see Materials and Methods). The first classifier used only the synchronization tempo, which captures the speed of the music (Figure 11A). The second classifier also contained ISI ratios at fractions of the synchronization tempo to capture information about the rhythm of the music that was present in the synchronized activity (Figure 11B). We combined the Ballroom and Songs datasets and randomly selected 75% of the stimuli for training the classifiers and 25% for testing, with 1000 re-samplings of training and testing trials.

**Figure 12:**
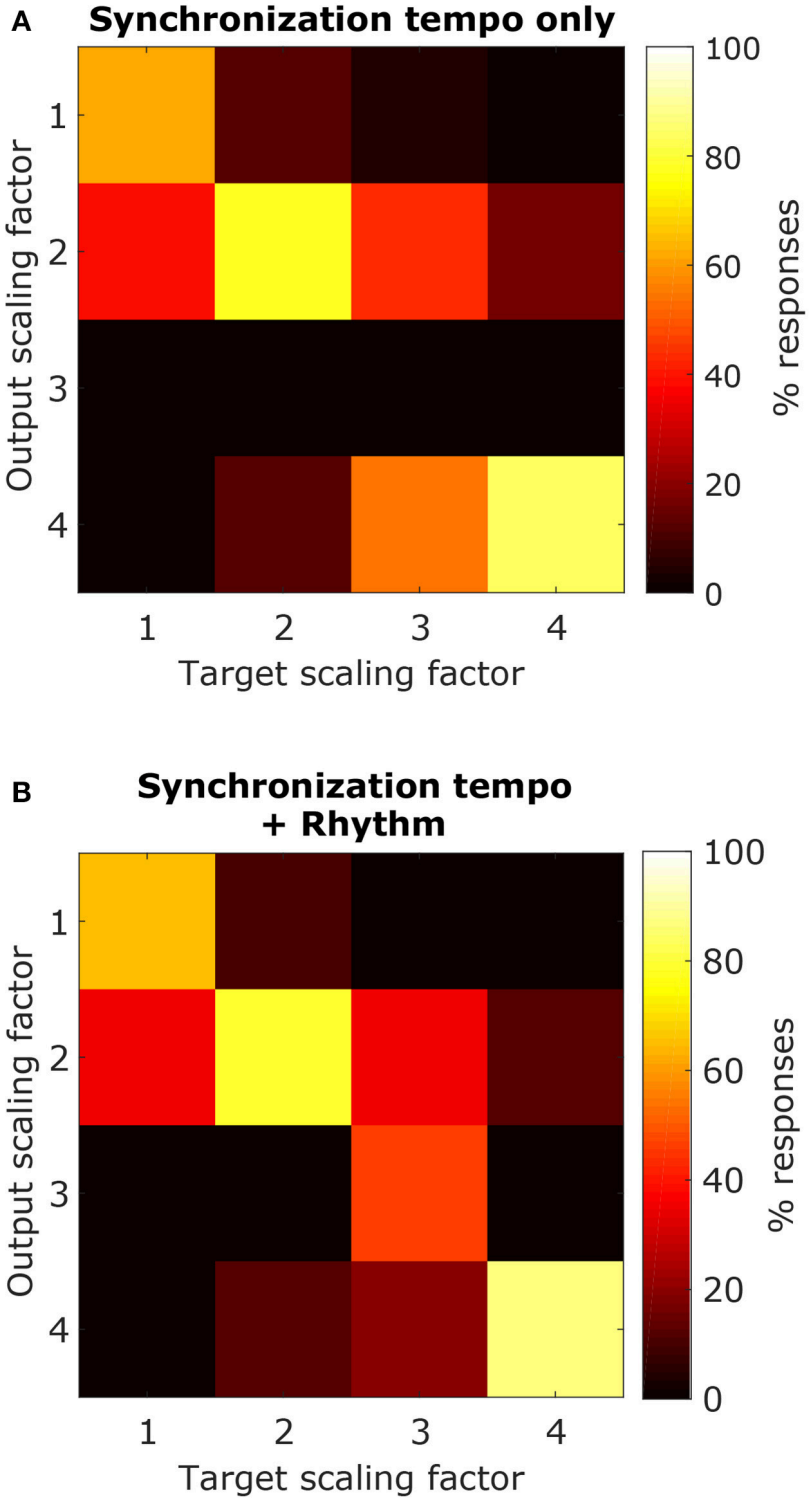
Confusion matrices for classifying each scaling factor with a classifier that just used the synchronization tempo **(A)** or a classifier that included the ISI ratios **(B)**. By including rhythm, there was an improvement in the classification of the 3x scaling factor but little improvement for classifying the other scaling factors.

Using the synchronization tempo alone, the scaling factor was classified correctly 72.3 ± 2.3% (mean ± standard deviation averaged across all re-samplings) of the time during testing. By dividing the synchronization tempo by the classified scaling factor, tempo-induction performance improved to 55.6 ± 2.5%. The classes were centered on synchronization tempos of 114 ± 2 BPM, 223 ± 2 BPM, 359 ± 13 BPM, and 397 ± 2 BPM for scaling factors 1–4, respectively. As expected, the class for the 1x scaling factor was centered on the 450–600 ms interonset interval range described for other music corpora from a previous study (van Noorden and Moelants, 1999) and the centers for the 2x and 4x scaling factor distributions were roughly twice and four times this range of intervals. The 3x scaling factor was never classified correctly and was often confused with the 2x and 4x classes (Figure 12A).

When rhythm information was included, the scaling factor was classified correctly for 76.4 ± 2.2% of the testing stimuli, and tempo-induction performance improved to 60.3 ± 2.6% (61.9 ± 3.3% for ballroom, 58.0 ± 4.1% for songs). The difference in performance between the two classifiers was only moderately significant (*p* = 0.016 for classification, *p* = 0.002 for tempo induction). The primary reason for the improvement in performance was due to an improvement in classification accuracy for the 3x scaling factor (Figure 12B). Thus, the perceived beat frequency may depend primarily on the speed of events, with a smaller contribution of rhythm.

## Discussion

In this study, we used models of the AN (Zilany et al., 2014), brainstem, and midbrain (Nelson and Carney, 2004; Carney et al., 2015) to simulate neural activity in response to isochronous sound sequences and real music. Our goal was to quantify tempo induction performance based on the simulated sub-cortical neural activity to directly identify the mechanisms necessary to “feel the beat” in music. Furthermore, by using a biomimetic model of acoustic processing in the brainstem and midbrain, we could identify specific additional stages of processing that are necessary to find the beat frequency of music. We found that midbrain-level processing, inherent randomness in neural activity, and a smoothing temporal window together limit the strength of neural synchronization to regular acoustic events and produce a preferred tempo around 100 BPM, in agreement with prior literature. Additionally, cochlear processing generates a low-audio-frequency bias for beat perception, but the tempi of the modulations themselves have a stronger effect on the synchronization tempo than the carrier frequencies. Lastly, despite these successes with simplistic acoustic stimuli, we found that the simulated neural activity often did not synchronize to the beat frequency, but instead synchronized to a multiple of the beat frequency. By using a classifier to appropriately scale the synchronization tempo to the actual beat frequency, tempo-induction performance improved considerably.

We found that midbrain model neurons with strong onset responses produced consistent preferred tempi around 100 BPM for clicks, SAM noise, SW noise, and tone pips. The SFIE model simulates synaptic mechanisms that could give rise to amplitude modulation tuning in the midbrain (Nelson and Carney, 2004). Alternatively, onset responses can also occur from adaptation mechanisms. Rajendran et al. (2017) showed that adaptation mechanisms in the midbrain of gerbils accentuate onsets in complex rhythms that could give rise to beat perception. However, the authors did not look at various envelope shapes. The responses of our model to these rhythmic stimuli for various event durations produced consistent vector strengths at the event frequency and variable vector strengths at all other possible tempi, and often the synchronization was strongest at the event frequency (Supplementary Figure 5), in agreement with our findings for musical recordings. If the events are short enough, we expect that adaptation mechanisms will accentuate the onsets of all events and could produce an equivalent result. Additionally, subjects vary regarding when they choose to tap to these stimuli (Nozaradan et al., 2012; Rajendran et al., 2017), which also suggests that the relationship between subcortical activity and the beat frequency is not one-to-one and may involve a learned mechanism that varies across subjects.

On average, cochlear processing in the AN fiber model appeared to produce a bias to low audio frequencies because the synchronization tempo was more often equal to the tempo for the tone pips with the low-frequency carrier. This bias provides a potential neurobiological reason for why low-frequency instruments carry the beat in some music (for example see Snyder and Krumhansl, 2001). However, it is tricky to test this perceptually; multiple instruments often play simultaneously on the beat, and cochlear delays can explain biases for simultaneous events (Wojtczak et al., 2017). Our stimuli used amplitude modulations at distinct tempi and phases to reduce the effects of simultaneous events, and we quantified the bias using the synchronization strength of neural activity rather than timing to specific events. The presence of a bias may be tested perceptually using these stimuli by having subjects either subjectively identify the beat of the stimulus or tap along with the stimulus at the beat frequency that they perceive. A crowdsourcing design may be most appropriate to properly sample the parameter space of these stimuli.

We used a temporal smoothing window to limit the upper range of tempi to 600 BPM based on previous work (Repp, 2005). This limit does not necessarily correspond to a peripheral motor limit because at this event rate musically trained participants are unable to accurately tap to every fourth event in a fast, isochronous sequence of acoustic events (Repp, 2003). For isochronous, simplistic stimuli, the temporal window was critical in producing the preferred tempo around 100 BPM in our model. However, we found that sub-cortical synchronization often occurred at a multiple of the tempo in musical recordings, and ultimately, by including a classification stage, tempo-induction performance was better without the temporal window. Then when is this temporal window applied? The temporal window defines a constant tolerance for detecting irregular events, but subjects can discriminate click rates around 10 Hz with an accuracy of about 3% (Ungan and Yagcioglu, 2014) implying that it cannot correspond to a limit in acoustic processing. Additionally, it is well known that cortical neurons can synchronize to acoustic periodicities at much faster rates (Joris et al., 2004). The window more likely corresponds to predictive tolerance rather than acoustic tolerance. The exact mechanism is unclear, but it could result from motor planning mechanisms that are used for tapping to regular events (Mendoza and Merchant, 2014; Patel and Iversen, 2014; Merchant et al., 2015; Merchant and Yarrow, 2016; Nozaradan et al., 2017). Motor synchronization may also affect the processing of regular acoustic events in the brainstem and midbrain (Nozaradan et al., 2016), and the accuracy of motor synchronization appears to be correlated with the temporal consistency of brainstem-level encoding of the speech syllable /da/ (Tierney and Kraus, 2013). However, in these studies, sub-cortical activity clearly synchronizes to the acoustics at frequencies higher than 10 Hz, so it is unlikely that the temporal window is applied in the brainstem or midbrain. To explain our findings for musical recordings in particular, it is more likely that temporal limitations are applied cortically and only after the beat frequency has been determined.

Our results suggest that the beat frequency cannot be determined based on the sub-cortical neural activity alone, and a second higher-level mechanism is necessary to perceive the beat. The importance of the relationship between the heard event frequency and the perceived beat frequency has been proposed in the past (London, 2012; Ding et al., 2017). It is unclear from our work what this mechanism might be; internal neural oscillators (Large et al., 2015), motor planning mechanisms (Patel and Iversen, 2014; Merchant et al., 2015; Merchant and Yarrow, 2016), or temporal coding of sequences in the hippocampus (Geiser et al., 2014) could produce patterns of neural activity at subharmonics of the synchronization tempo. However, the process of going from the neural synchronization tempo to the actual tempo is likely to involve a dynamic, high-level system. Listeners can change where they perceive the beat for stimuli with identical rhythms (Iversen et al., 2009). One's preference for the location of the beat is based on experience, since beat perception varies with culture (Drake and El Heni, 2003) and infants prefer different beat frequencies for identical stimuli depending upon the frequency of vestibular sensation during training (Phillips-Silver and Trainor, 2005). Lastly, whereas people often agree on a particular beat for a piece of music, people may tap individually to music at different frequencies and phases relative to the expected tempo (McKinney and Moelants, 2006; Patel and Iversen, 2014). Thus, the relationship between the event frequency and the beat frequency is likely learned through experience and is not due to an innate mechanism.

The techniques used in our modeling work are similar to those used in other algorithms for tempo induction, but our model is unique in predicting the tempo of music using biomimetic models of sub-cortical auditory processing. Several tempo-induction algorithms introduce a template-matching stage that determines the proximity of the computed onset histogram for a single clip of music to a database of onset histogram templates for different rhythms (Seyerlehner et al., 2007; Holzapfel and Stylianou, 2009). Elowsson and Friberg (2015) also included the “speed” of the music, which was determined by a weighted average of the two most probable tempi for the song. In their implementation, both the rhythm information and the speed were used as inputs to a logistic classifier that ultimately determined the tempo (see also Levy, 2011 for the importance of speed judgments in tempo induction algorithms). Our classification scheme is similar. We show that a classifier based on the “speed” alone (the synchronization tempo) does well at identifying the appropriate scaling factor for determining the tempo. We also found that the pattern of interspike intervals, which was used to quantify rhythm, provides a small, albeit significant, amount of information for tempo induction. Also, in our implementation, we assumed that beats are determined based on the summed activity across CF. Similar algorithms detect onsets when the energies in multiple audio frequency bands peak simultaneously (Scheirer, 1998; Klapuri et al., 2006; Ellis, 2007). In contrast, other algorithms have used the frequency content to categorize onset events (Elowsson and Friberg, 2015; Krebs et al., 2016). It is clear that the auditory system combines frequency content into discrete events (Bregman, 1990; Darwin, 1997; Shamma et al., 2011), but where this combination occurs relative to beat processing is unclear. Nevertheless, our model might improve in performance if we introduce a stage that isolates cross-CF neural activity into discrete temporal objects and identifies the tempo based on the pattern of objects rather than on the summed neural activity alone.

Our technique inherently assumes that events equally divide beats and the rhythm that results is based on small integer ratios, which is true for the songs in the datasets we used. However, there are some songs in which the beat of the music is not isochronous, particularly when the music has a complex meter (London, 1995). Our model will identify the regular intervals of events in this case, but a more complex learning mechanism that can identify the explicit timing of non-isochronous beats would be necessary for these particular applications. More strikingly, in Malian jembe drumming, events do not occur at integer ratio subdivisions of the beat (Polak et al., 2016). Music with more complex subdivisions of the beat is particularly problematic for our model because it relies on the initial identification of an event frequency. The issue can be resolved, however, by recognizing that humans have a fairly high tolerance for deviations from synchrony when listening to regular events (Repp and Penel, 2002). The drumming is produced with consistent offsets from the isochronous subdivisions of the beat but they may still be within our perceptual tolerance to asynchrony. A similar effect is observed in classical music; performers slightly vary the timing of notes relative to the strict note durations of the piece for expressive purposes (for review see Patel, 2010). If perceptual processes and motor processes can distinctly subdivide beats, then non-musicians in Mali might subdivide isochronous intervals more evenly than jembe musicians who have experience reproducing the non-isochronous events in the music (see Jacoby and McDermott, 2017).

Our results demonstrate the importance of using real music to study beat perception. Previous studies have primarily used acoustically salient events with complex rhythms. We have shown that the speed of events is relatively more important for tempo induction than the rhythm of those events in musical recordings. We encourage other groups to study the perception of rhythm with biomimetic models of the auditory system. We also encourage others to use real music as stimuli, since musical recordings provide more realistic conditions by which we can better understand how the human brain processes music in general.

## Author contributions

NZ designed, performed the study, analyzed the data, and wrote the manuscript. NZ, LC, and EL interpreted the results and approved the final version of the manuscript.

### Conflict of interest statement

The authors declare that the research was conducted in the absence of any commercial or financial relationships that could be construed as a potential conflict of interest.
