# Supplementary material for: Preferred Tempo and Low-Audio-Frequency Bias Emerge From Simulated Sub-cortical Processing of Sounds With a Musical Beat
Source: Front Neurosci. 2018 May 29;12:349. doi: 10.3389/fnins.2018.00349 (PMC5987030; doi:10.3389/fnins.2018.00349)
Supplement: Supplementary file 1 [file DataSheet_1.docx]

***Supplementary figures***

**Preferred tempo and low-audio-frequency bias emerge from simulated sub-cortical processing of sounds with a musical beat**

Nathaniel J. Zuk*, Laurel H. Carney, Edmund C. Lalor

***Correspondence:** Corresponding author: nzuk@ur.rochester.edu


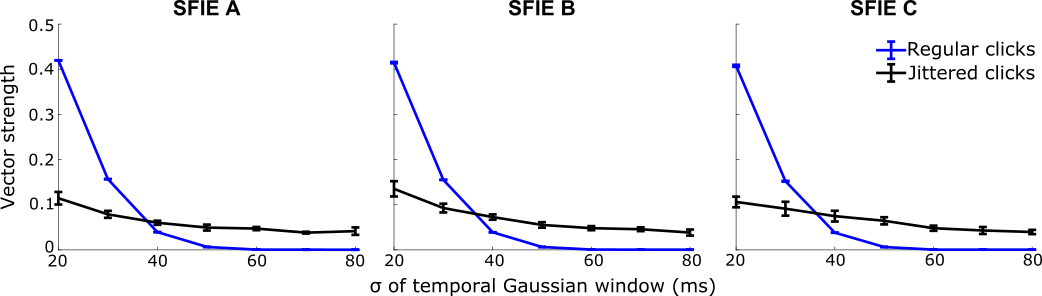


**Figure 1**: The stimuli were 1 ms clicks at an average rate of 600 BPM (see Methods in the manuscript) with either isochronous (regular) or 100% jittered timing. After simulating subcortical neural activity (Figure 1 of the manuscript), the standard deviation of the temporal smoothing window was varied between 20 – 80 ms in 10 ms steps (Figure 3a of the manuscript). Each stimulus was presented 10 times. For the jittered clicks the timing of the clicks was randomized for each trial. Error bars designate interquartile ranges. We selected a standard deviation of 40 ms because, at this width, the vector strength for regular clicks consistently fell below the vector strength for 100% jittered clicks irrespective of the SFIE model used.


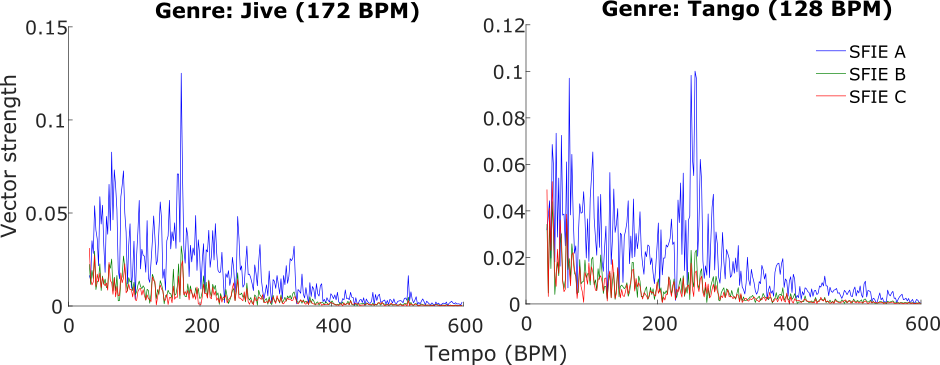


**Figure 2**: Vector strengths for two example musical recordings from the Ballroom dataset (SFIE A: blue, SFIE B: green, SFIE C: red). The vector strengths were computed after the temporal Gaussian window was applied. The dance genres and ground truth tempos for each are shown above. Of the three SFIE models, SFIE A produced the largest vector strengths for musical recordings.


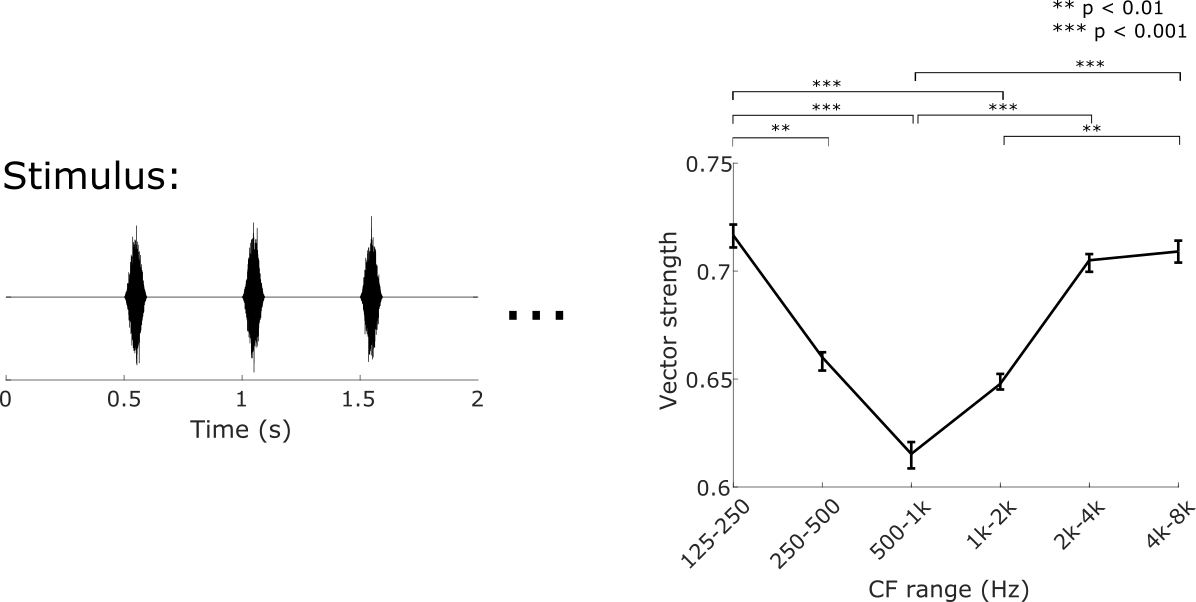


**Figure 3**: For stimuli consisting of tone pips at different frequencies and different tempos, synchronization tempos were more often closer to tempo for the tone pips with the low-frequency carrier, thus demonstrating a low-audio-frequency bias for tempo induction. The low-audio-frequency bias could be due to an increased spread of excitation along the basilar membrane for low frequency stimuli (Hove et al, 2014). Alternatively, AN fibers at different CF ranges have different response patterns (for example, low-CF fibers “ring” for very brief broadband stimuli) and this could also contribute to the bias (Kiang et al, 1965; Zhang et al, 2001).

To examine the effect of CF range on the low-audio-frequency bias while minimizing the effect of the spread of excitation, stimuli consisted of broadband noise modulated using 100 ms raised-sine “pips”, which was identical to the modulations for the tone pips. The tempo of the stimulus was always 120 BPM, the duration was 10 seconds, and each stimulus was repeated for 10 trials using a different noise token each trial. The range of AN fiber CFs was then varied, and 20 AN fibers, logarithmically spaced in CF, were used per one octave range. If AN fiber response differences contribute to a low-audio-frequency bias, then the vector strength for these broadband stimuli should be higher at low CFs than at high CFs.

The plot shows median vector strengths and interquartile ranges for each CF range. There was a significant variation in vector strength across CF range (Kruskal-Wallis test: χ^2^ = 54, p < 0.001). With a multiple comparisons test using Tukey’s honest significant difference, the middle CF range was significantly different than the upper and lower ranges. However, there was no significant difference between the vector strengths for the low CF ranges and the high CF ranges. For the stimuli with two tone pips, the strongest biasing occurred when the two carrier frequencies of the tone pips were the furthest apart. The findings based on CF ranges alone cannot explain that result. Thus, we think that the low-audio-frequency bias we observed was due to the spread of excitation in the basilar membrane.


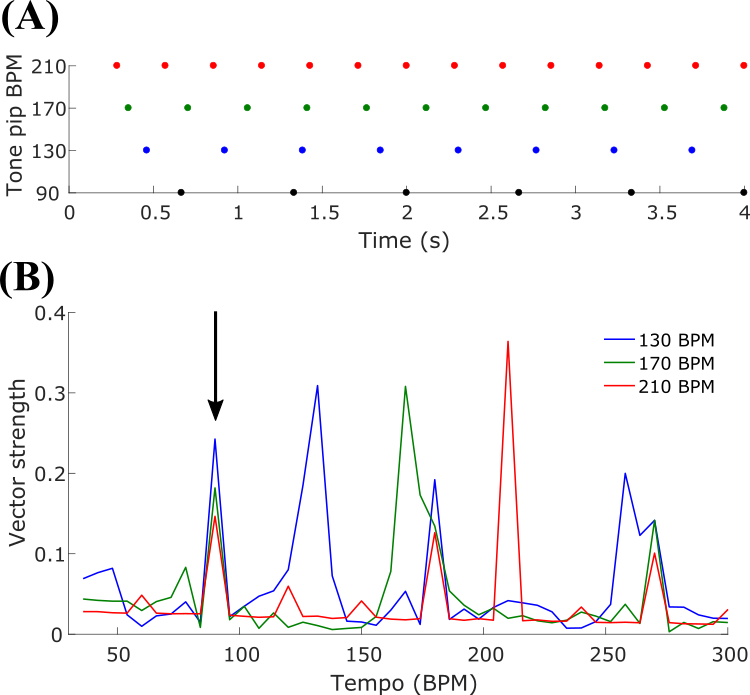


**Figure 4**: Demonstration of the change in vector strengths for the two tone-pip tempos as the upper tone-pip tempo increases. Stimuli consisted of two tone pips: f_L_ = 250 Hz, T_L_ = 90 BPM, f_H_ = 1 kHz, and T_H_ could be either 130, 170, or 210 BPM. The exact timing of the different tone pips is shown in **(A)**. The black tone pips at 90 BPM were always presented, and either the blue (130 BPM), green (170 BPM), or red (210 BPM) tone pips were presented simultaneously with the black tone pips. **(B)** The vector strengths as a function of tempo for the three different stimuli after averaging across 10 repetitions of each stimulus. The vector strength for the faster tempo increases as the tempo increases from 130 BPM to 210 BPM. Additionally, there is a suppression of the vector strength at 90 BPM (black arrow) as the tempo of the other tone pips increases from 130 to 210 BPM.


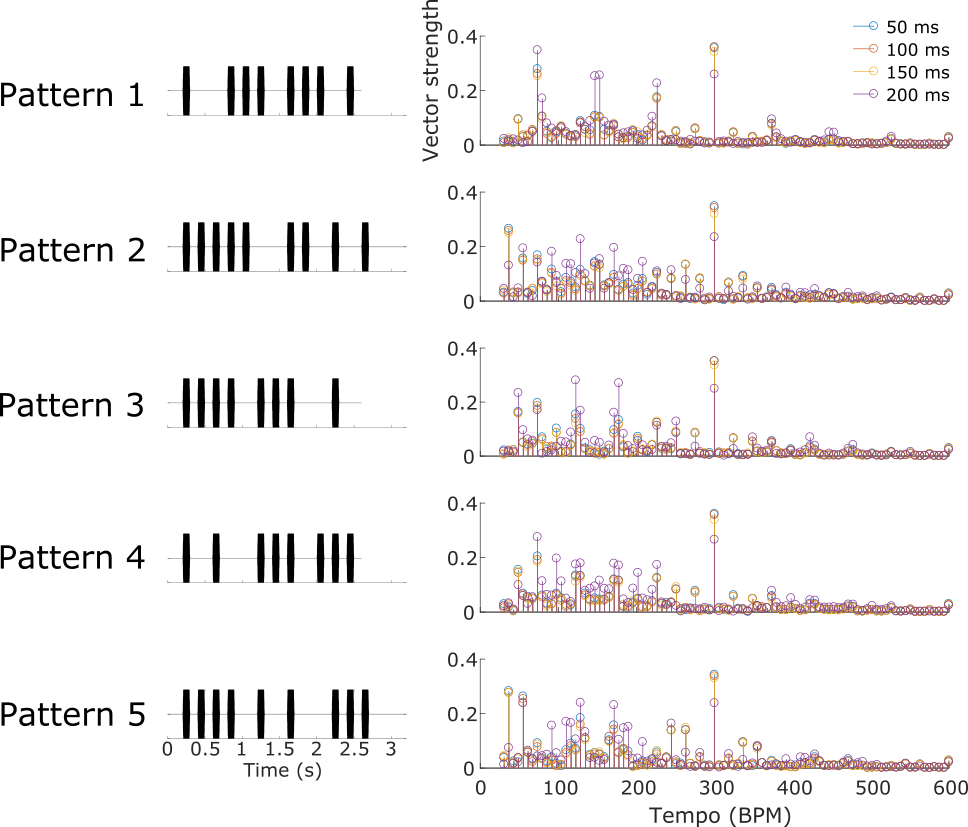


**Figure 5**: Stimuli consisted of 5 complex rhythmic patterns used previously to study the neural encoding of beats and beat perception (Henry et al, 2017; Nozaradan et al, 2016; Nozaradan et al, 2012; Rajendran et al, 2017). Each event was a continuous 990 Hz tone with a 10 ms raised-sine onset and offset ramp, and the event duration was varied from 50 to 200 ms (Henry et al, 2017). The event rate of all stimuli was 300 BPM, equivalent to an inter-event interval of 200 ms. Each stimulus consisted of 200 ms of silence followed by 9.6 s of the repeating pattern (patterns 1, 3, and 4 were repeated 4 times, patterns 2 and 5 were repeated 3 times). The temporal smoothing window was applied to the PSTH before computing the vector strength of each stimulus. The vector strengths on the right have been averaged across 10 trials of each stimulus.

One cycle of each pattern is shown on the left, and the corresponding vector strengths for simulated response are shown on the right. Responses for different event durations are shown overlapping in the stem plot. Vector strength values were very consistent across trials; the maximum standard deviation for any of the vector strengths was 0.002. The vector strengths at the event frequency, 300 BPM, remain very consistent irrespective of the pattern presented. For event durations of 50-150 ms, the synchronization tempo was always 300 BPM. For the event duration of 200 ms, the synchronization tempo was 300 BPM for pattern 2 and between 75-200 BPM for all other patterns.

Based on our findings for musical recordings, we think that the synchronization at 300 BPM is used to determine the beat of the rhythmic patterns. The stability of the vector strength at this tempo is consistent with the perceptual constancy of the strength of the beat when the event duration is varied (Henry et al., 2017).

**References**:

Henry, M. J., Herrman, B., & Grahn, J. A. (2017). What can we learn about beat perception by comparing brain signals and stimulus envelopes? *PLoS ONE*, *12*(2), e0172454. doi: 10.1371/journal.pone.0172454

Hove, M. J., Marie, C., Bruce, I. C., & Trainor, L. J. (2014). Superior time perception for lower musical pitch explains why bass-ranged instruments lay down musical rhythms. *Proc. Natl. Acad. Sci. U.S.A.*, *111*(28), 10383–8. doi: 10.1073/pnas.1402039111

Kiang, N. Y.-S., Watanabe, T., Thomas, E. C., & Clark, L. F. (1965). *Discharge Patterns of Single Fibers in the Cat’s Auditory Nerve.*

Nozaradan, S., Peretz, I., & Keller, P. E. (2016). Individual Differences in Rhythmic Cortical Entrainment Correlate with Predictive Behavior in Sensorimotor Synchronization. *Sci. Rep.*, *6*, 20612. doi: 10.1038/srep20612

Nozaradan, S., Peretz, I., & Mouraux, A. (2012). Selective neuronal entrainment to the beat and meter embedded in a musical rhythm. *J. Neurosci.*, *32*(49), 17572–17581. doi: 10.1523/JNEUROSCI.3203-12.2012

Rajendran, V. G., Harper, N. S., Garcia-Lazaro, J. A., Lesica, N. A., & Schnupp, J. W. H. (2017). Midbrain adaptation may set the stage for the perception of musical beat. *Proc. Biol. Sci.,* *284*(1866), 20171455. doi: 10.1098/rspb.2017.1455

Zhang, X., Heinz, M. G., Bruce, I. C., & Carney, L. H. (2001). A phenomenological model for the responses of auditory-nerve fibers: I. Nonlinear tuning with compression and suppression. *J. Acoust. Soc. Am.*, *109*(2), 648–670. doi: 10.1121/1.1336503
